# Bringing prosocial values to translational, disease-specific stem cell research

**DOI:** 10.1186/1472-6939-15-16

**Published:** 2014-02-27

**Authors:** Reuben G Sass

**Affiliations:** 1 Case Western Reserve University, 10900 Euclid Avenue, Cleveland, OH 44106-4976, USA

**Keywords:** Stem cells, Communitarianism, Cultural studies, Patient advocacy, Moral philosophy, Behavioral economics

## Abstract

**Background:**

Disease-specific stem cell therapies, created from induced pluripotent stem cell lines containing the genetic defects responsible for a particular disease, have the potential to revolutionize the treatment of refractory chronic diseases. Given their capacity to differentiate into any human cell type, these cell lines might be reprogrammed to correct a disease-causing genetic defect in any tissue or organ, in addition to offering a more clinically realistic model for testing new drugs and studying disease mechanisms. Clinical translation of these therapies provides an opportunity to design a more systematic, accessible and patient-influenced model for the delivery of medically innovative treatments to chronically ill patients.

**Discussion:**

I focus on disease-specific cell therapies because the types of patients who would benefit from them have congenital, severe, high-maintenance chronic conditions. They accordingly have a very strong claim for medical need and therapeutic intervention, must interact regularly with health providers, and so have the greatest stake in influencing, at a systemic level, the way their care is delivered. Given such patients’ shared, aggregate needs for societal support and access to medical innovation, they constitute “patient communities”. To reify the relevance of patient communities within a clinical context, I propose competitive grants or “prizes” to spur innovation in delivery of care, promoting “prosocial” values of transparency, equity, patient empowerment, and patient-provider and inter-institutional collaboration. As facilitators of participant-driven advocacy for health and quality of life-improving measures, patient communities may be synergistic with the broad-based, geo-culturally embedded public health networks typically referred to as “communities” in the public health literature.

**Summary:**

Prosocial values acquire a strong ethical justification based on shared need, and can be clearly defined as grant criteria, when applied to patients such as those who will benefit from disease-specific stem cell treatments. Within this context, prosociality aims not just to expand patients’ treatment choices, but also their opportunities to take a more active role in the management of their own care and contribute towards shared goals through better-informed advocacy. Accordingly, prosociality promotes relational autonomy as well as other basic bioethical principles, including beneficence and a holistic, relational conception of human dignity.

## Background

When we talk about medical innovation, we are typically referring to new drug treatments or therapies. In recent years, however, stem cells have come to represent an archetypal form of medical innovation—their potential to differentiate and repopulate any cell type raises the possibility of a paradigm shift in medical therapy, based on a largely untapped but theoretically almost limitless promise of regeneration [1,2]. But what about innovation in *delivery of care* for the patients with chronic conditions who will likely benefit from such therapies?

This article describes an incentive system, and provides an ethical basis, for a more systematic, accessible, and patient-influenced approach to delivery of medically innovative stem-cell treatments, embodying a concept which I refer to as “prosociality” [3]. The aim is to show that a patient community can amount to more than a mere logistical convenience, a means of expediently grouping together clumps of patients for purposes of insurance billing or clinical data collection. A prosocial system is ethically premised on the recognition of patients’ shared, aggregate *needs* for societal support, entailing the ethical importance and indeed essentialness of the concept of a community of patients. Consequently, incentives which seek to improve both patient care and experience should be more relationally focused and patient-directed. Indeed, a *community of patients* with a given chronic condition can serve as a mediator and a source of empowerment for its members, who must negotiate their care within the world of health care.

While the attendant requirement of aggregate, shared, and consistent need results in a strict definition of a patient community, this is in no way intended to introduce a concept in opposition to the kind of broad-based, geo-culturally embedded public health network which is typically what the public health literature refers to as a “community”. Indeed, as explained later, public health networks (as well as regulatory or grant-making bodies on which patient advocates are represented) can serve as an interface through which more strictly defined patient communities can interact with more broad-based groups or coalitions, advocating together for health and quality-of-life-improving measures.

This article focuses on disease-specific iPS cell therapies because the types of patients who would benefit from them have congenital, severe, high-maintenance chronic conditions (such as Fanconi Anemia, Gaucher Disease, Spinal Muscular Atrophy, and Severe Congenital Immunodeficiency Syndrome). They accordingly have a very strong claim for medical need and therapeutic intervention, must necessarily interact regularly with health providers, and so have the greatest stake in influencing, at a *systemic level*, the way their care is delivered. Moreover, the emergence and clinical testing of disease-specific stem-cell therapies offers an opportunity to design a new system for delivery of medically innovative treatments to patients with chronic conditions, especially given the scale of societal resources involved in their development. In the U.S., NIH (the National Institutes of Health) distributed nearly $1.5 billion for embryonic and non-embryonic stem cell research in 2012 alone, to say nothing of the manpower used and information collected [4].

Indeed, disease-specific stem cell therapies, created from cell lines containing the genetic defects responsible for a particular disease, have the potential to revolutionize the treatment of refractory chronic diseases. Given their capacity to differentiate into any human cell type, these cell lines might be reprogrammed to correct a disease-causing genetic defect in any tissue or organ, in addition to offering a more clinically realistic model for testing new drugs and studying disease mechanisms [5,6].^a^ Relevant to the focus on patient communities, in the U.S. virtually all of the diseases which are the subject of iPS research are also associated with eponymous foundations (e.g. the Fanconi Anemia Fund and Spinal Muscular Atrophy Foundation), which support research for their respective diseases.

The present proposal would thus require optimizing, at a community level, the care delivery experience for patients with the types of chronic diseases which are the subject of disease-specific stem-cell research, so as to facilitate patients’ equitable access to therapies, engagement in advocacy, research participation, and awareness of the treatment options and clinical and quality-of-life implications of their conditions (consistent with patients’ or guardians’ preferences and informed consent). Such a system would ensure that when the benefits of stem cell research materialize on a larger clinical scale, they do so in such a way as to reify the concept of the patient community as an organizing principle for health care, via four core values—transparency, equity, patient empowerment, and inter-institutional and patient-provider collaboration. The value of equity is particularly critical within the context of disease-specific stem-cell therapies, given that they are highly specialized biologics which tend to be much more expensive than standard primary care services. Altogether, these principles complement and partially overlap the social justice goals of the International Society for Stem Cell Research [7]. No guidelines either proposed or implemented, however, distinguish them within a shared context, apply them to translational iPS research, or explain how they might operate synergistically by strengthening patient communities.

This discussion is not solely concerned with how providers should interact with individual patients, as is much of the bioethical literature. The proposal does not just seek to incentivize the care delivery system to be patient-centered in terms of a top-down respect by providers and institutions for patient autonomy. Perhaps more consequentially, it would aim to cultivate patient communities as important stakeholder groups, with more scope to dynamically influence policy at the institutional and perhaps even the regulatory level. Stem-cell research involves a broad array of societal stakeholders, including regulatory decisionmakers, translational researchers in academia, and private companies. The utimate goal is to establish a more rhizomatic structure for patient-provider and patient-society interaction, one which would afford opportunities for lateral, less hierarchical, and more closely intertwined channels of patient feedback which can exert meaningful influence on patient care. Should regulators, medical leaders and funders choose to embrace the values underlying my proposal, my hope is that medical centers would re-orient care delivery so as to interact with patients at the level of patient communities, better positioning all patients to actively address their shared medical needs and achieve shared goals —improving quality-of-life by improving health, and promoting relational autonomy through collective engagement.

Borrowing from proposals for promoting innovation in medical technology, criteria are set forth for operationalizing these values via competitive grants or prizes targeted toward academic medical centers (perhaps in partnership with community health practices). Competitive grants for *care delivery* are inspired “prizes” which aim to spur innovation in health care and eco-sustainable technology. Such prizes may be offered by for-profit or not-for-profit entities, and even constitute the basis for a proposed alternative to the United States’ existing patent system, via the Medical Innovation Prize Act [8].^b^ The goal is to incentivize these centers to develop, test, and refine strategies for a prosocial model of *care delivery*. These principles, when working in tandem as embodied in the patient community, not only do not infringe on autonomy, but promote both it and other basic bioethical values, including beneficence and bioethicist Charles Foster’s holistic, relational conception of human dignity [9].

## Discussion

### Bringing prosocial values to translational iPS cell research

In the U.S., such patient communities, often via local chapters of disease-specific foundations, are already active at the national or local level in policy advocacy and campaigns for research funding, and would benefit alike from developments in disease-specific stem cell therapies. In addition to the potential for government funding or public-private partnerships, foundations may be able to partner with each other to fund such grants, which could take into account a broad subset of chronic diseases treated at academic medical centers. Various metrics which are suggested for grantmaking, as well as the very existence of the grants, might encourage the implementation of the core principles of prosociality—transparency, equity, patient empowerment, and collaboration—which are altogether broadly consistent with the International Society for Stem Cell Research Guidelines for Clinical Translation [7]. At the most basic level, the collection and publication of data relevant to the awarding of grants will promote transparency.

Equity is also critically relevant, given the high cost of stem-cell based therapies and their highly concentrated development and translational testing at certain highly specialized academic medical centers. The high expense of stem cell therapies, and related concerns about equity and access, stem from their speculative nature, the degree of technical and laboratory manipulation involved in their development and production, and the need for companies to earn a financial return on the investment costs and decades’ worth of research necessary to develop efficacious and safe products. Currently an allogeneic bone marrow transplant can cost up to $200,000 in the U.S. [10]. To assess the equity of translational research and clinical care delivery, it is essential to have access to the demographic and socio-economic composition of stem cell research participants, or those receiving experimental therapies, as recommended by the ISSCR [7]. In the U.S. a comparison of the percentage of Medicaid vs. non-Medicaid ^c^ participants might be helpful. In any case, nuanced analysis of data might allow for the identification of underserved patients in particular demographic or SES categories, and accordingly grant points could be awarded for measures to improve access among these patients, to the extent medically appropriate and logistically feasible. In addition to promoting equity, systemwide analysis of patterns of enrollment in clinical trials or access to therapies, stratified by medical condition and aligned with degree and urgency of medical need, appropriateness of intervention, etc. could serve as broader measures of implementation. Overall, the grants would incentivize, at the level of institutional policy, improved awareness among patients of clinical trials and treatment options, knowledge of their condition and its clinical implications (should they wish to know), and opportunities for collaborating, supporting and sharing experiences with other patients and families with their condition, via support groups or advocacy. In the subsequent discussion of the proposal’s autonomy-enhancing potential, these qualities are referred to as “patient empowerment”.

Indeed, grant criteria could measure the extent to which patient support groups can attract provider participation and integrate with medical centers’ existing supportive care programs (e.g. for counseling, coping skills, and logistical aid), partner with foundations and advocacy organizations, and offer opportunities for patient-submitted feedback. This would foster collaboration among patients with the same condition (members of the same patient community), as well as between patients and providers. To promote both inter-institutional collaboration and equity, I recommend that institutions also receive grant points for sending patients to *other* centers which offer clinical trials, therapies, or procedures for which the referred patients might be good candidates. Patient satisfaction or awareness surveys might help determine patients’ perception of the adequacy of their access to information about clinical trials, daily coping methods, or experimental therapies (this last being most relevant in life-critical situations). Standardizing such surveys would allow for fair and consistent comparison of medical centers, and could be accomplished with input from a broad range of centers. Indeed, some measures of *patient experience,* not just *clinical outcomes*, must be available. By developing grants contingent on all these measures and publishing the data, providers would be encouraged to make the sharing of such information a matter of routine practice.

The ISSCR further notes that “access [to stem cell therapies] will depend on financial terms and business models that are perceived as fair by all stakeholders, including patients, providers, payers, companies and governments” [7]. By contributing to a better-informed patient community—more active in policy advocacy and the management of their care, and with more options and resources for communicating and collaborating with patient advocates in decisionmaking roles—the present proposal would thus advance the ISSCR’s goal of fostering a more inclusive public discussion with a greater influence on policy outcomes. Grant criteria could further improve transparency by encouraging the publication, for public benefit, of data on experimental therapies for individuals (which, if successful, would hopefully suggest a basis for future trials), as recommended by the ISSCR [7].

### Defining the community of needs

Much of the recent literature in health policy and public health recommends that care delivery systems should adopt a population-level or community focus in trying to optimize health outcomes [11,12]. Indeed, few studies have analyzed, from a cultural or bioethical perspective, what constitutes a community of patients within the healthcare system, or what ethical implications such categorizations of community have for care delivery. Having discussed the operational criteria for a prosocial delivery model, exemplified in the proposed grants, it is important to justify the indispensable role of the patient community in shaping, ethically and practically, the identities of patients with chronic diseases, the nature of their interactions with each other and their providers, and the claims they make on societal resources channeled through the health care system (including those flowing from the government agencies and private foundations).

First, we must consider the scope of community. From the perspective of a patient with a chronic condition, we can conceive of the health care system as a series of overlapping, nested communities. An academic medical center is the geographical locus for a community of patients with a given condition who receive treatment at that center. Members of this local community may receive treatment from the same physician, belong to the same patient and family support groups, and volunteer for or donate to the same disease-specific foundation. Thus members of a local patient community interact with each other face-to-face, sharing first-hand information about treatment experiences, health status and life challenges, and joining together at the same fundraising or advocacy events. Such interactions create what Mitchell refers to as “local and visible links”, since individuals’ knowledge of their neighbors’ well-being comes from what they see and hear person-to-person rather than from impersonal, anonymized statistics [13]. We can also apply the notion of a patient community in two broader senses: as encompassing all the patients nationally or internationally who have a given chronic condition, as well as the sum total of patients with a variety of chronic conditions at a given academic medical center or network of community health practices. Of course, both the providers and chronically ill patients at an academic medical center have profoundly personal interests in the center’s research agenda and capacity to deliver innovative treatments and procedures when no adequate alternative exists. Nevertheless, in this analysis providers constitute a stakeholder group distinct from patient communities, unless they suffer from the symptoms of a given chronic disease or care for family members who do (in which case their membership would stem from their capacity as patients or caregivers).

As with patient communities, the family unit is another sphere of community which creates, according to some communitarian theorists, inherent entanglements between its members, resulting in shared interests and obligations [14]. In health care, of course, patients, patient communities and family units interact, in both policy advocacy and treatment decisions, but the role of parents or siblings in a patient community is not necessarily fixed. Indeed, the nature and degree of families’ involvement depends on patient age and condition, in addition to family-specific factors, such as family composition and belief and value systems. Parents and siblings would be much less involved, in time, money, and emotional investment, in the care of a 40-year old self-sufficient patient with type I (Juvenile) diabetes than they would be for a 2-month old Severe Combined Immunodeficiency patient who needs an HLA-matched bone marrow transplant from a sibling. (Both of these diseases are the subject of disease-specific iPS research). So while families may have an intrinsic interest in related patients’ well-being—at least assuming that parents are competent and siblings are willing to offer some support for each other—they should only be considered members of patient communities if they are primarily responsible for the patient’s health, are heavily committed in time and energy to caretaking, or are very active in health advocacy, raising awareness of patients’ concerns among providers, researchers, and policymakers. Of these three conditions, the first would carry the greatest weight. Even so, families’ involvement in such communities should not automatically give them an influence over treatment decisions equal to that of mentally competent adult patients. For family members without proxy, involvement in patient communities would instead indicate their commitment to—and a corresponding interest in—improving patients’ quality of life and directing societal resources towards their care, so as to reduce the overall social burden of the disease.

So what is the nature of the community bonds between patients with the same chronic condition, and what are the ethical implications for delivery of care? The interests of such patients are fundamentally aligned—they share the same medical challenges, which pose a similar threat to quality of life, and they have a shared interest in securing a broad-based, well-funded, and well-staffed research agenda for their condition. Families may likewise share similar caretaking burdens. The toll exacted by chronic conditions on patients and families is well-suited for empirical assessment—whether in terms of mortality, stress level, life satisfaction, absenteeism from work or school, etc. Patients have a shared interest, often via the same therapeutic means, of overcoming the burden of their condition, as reflected by a score as close as possible to optimal on such metrics. Of course, “optimal” can be a context-dependent designation, mediated, within a particular patient-physician-caregiver relationship, by criteria including specific sociocultural influences, family structure and the role of familial interactions, and the patient’s functional limitations—the last of which it is of course the particular job of medication and procedures to mitigate. To the extent that all chronic conditions cause adverse physiological symptoms, reduce quality of life, and interfere with patients’ ability to live emotionally fulfilling lives while participating fully and unrestrictedly in the activities of their choice—in school, work, family life, recreation and exercise—all patients whose conditions cannot be cured or adequately controlled suffer harm, and those with the same condition and treatment options suffer a similar type of harm. In philosopher David Wiggins’s analytic definition, if a lack of *x* necessarily causes one harm*—*with reference to some objective “standard of flourishing,” and given the stipulation that one’s behavior remains “morally and socially acceptable” under circumstances which are realistically conceivable (economically, physically, technologically, culturally, etc.)—then one has a *need* for x, even if one does not know it [15]. Admittedly the degree of harm as well as the therapeutic options vary based on condition. For example, regular insulin injections may be sufficient to secure a normal quality-of-life for many patients with type I diabetes. The only viable option for SCID, however, is a bone marrow transplant, and type 1 spinal muscular atrophy (SMA), typically fatal by the age of two, lacks any treatment options at all, making advances in iPS and gene therapy all the more vital for SMA patients [6,16]. As Wiggins himself acknowledges, the notion of harm can vary based on sociocultural context. But a distinguishing characteristic of many severe chronic conditions (e.g. muscular dystrophy, SCID, Fanconi Anemia, SMA, Gaucher disease type III, Huntington’s Disease) which are the subject of iPS research is that they are congenital and their underlying symptoms—the primary health dangers and risks of the disease—cannot be adequately addressed without (in many cases undeveloped) pharmacological, genetic, or stem cell-based interventions [17]. In the health care context, such congenital genetic disorders with severe or life-threatening symptoms are perhaps the strongest candidates for an undeniably basic need—one whose basicness stems from “laws of nature, unalterable and invariable environmental facts, or facts about human constitution” [15]. The lack of any alternative therapies for addressing the need, and the constant fact of not being able to fully actualize and re-develop one’s life goals without treatment, may add to the need’s *vitalness* (to use Wiggins’s terminology), reinforcing the strength and rigor of the need claim.

### Defending the ethical relevance of patient communities

From a libertarian perspective, Wiggins suggests that “one aspect of [need’s] indispensability concerns the defense it can afford to an individual, in a community that has found a way of adjusting its institutions to its sentiments…” [15]. But in contrast to a conception of need as a justification for an individual right which protects against community encroachment, the interests of patients with the same chronic condition may be so closely aligned as to constitute an *aggregate, consistent, shared* need that can be defined in very rigorous scientific terms. Such aggregate needs have the same moral force as individual needs. But because these needs encompass large groups and, in the context of iPS therapy, potential remedies require a very high degree of technical sophistication, manpower, and information, the duty to furnish these resources falls on societal institutions and regulatory authorities. Indeed, within the sphere of health care, beneficence is specially obligatory. In this way, societal measures which recognize the collective importance of addressing these shared needs are justified. Such measures include insurance subsidies and regulations—in particular, in the U.S., the Affordable Care Act’s ban on coverage exclusions for pre-existing conditions, and its elimination of lifetime limits (“caps”) on the benefit payouts for health insurance policies—society has assumed an explicit role in validating the needs of those with chronic conditions and removing barriers to accessing the objects of their need, i.e. medical therapies [18]. Moreover, the high priority which society places on the future therapeutic value and scientific innovation embodied by stem cells is evident in the massive amount of NIH grants distributed for embryonic and non-embryonic stem cell research in 2012 alone [4]. Such social policies and regulatory priorities, justified on the basis of shared needs, greatly facilitate the non-profit action necessary for the accessible, community-focused care delivery system defended here.

When societal resources (of money and knowledge and manpower)—whether channeled directly through government, or non-profit research institutions and insurers—flow towards such *shared needs*, they result in a form biosocial regulation whose objects, at the policy level, are not individuals but communities of patients who share a need for access to a broader, more effective range of therapies. Because patients’ identities intersect with their membership in a community based on such a shared need, their collective satisfaction of that need must be defined in aggregate terms. It must stem from networks of providers and research institutions which consider patient communities central to their mission, not only in order to translate research advances into therapies which address the most clinically serious problems facing the community, but to deliver those therapies in such a way as to maximize, given the extent of available societal resources, the overall quality of life and prognosis for those living with the disease. This contrasts with a neoliberal model, where individuals or entities are divested of all communal linkages and the question is: how can I devise the most profitable means of giving individual customers a means of speculating on their own disparate biological futures for their personal benefit? Instead, the question we should ask is: given society’s interest in addressing the shared needs of patient communities, how can we incentivize the delivery of innovative treatments and stem-cell therapies so as to give patients the assurance that society will use its resources as efficiently as possible to minimize the harm that their disease can cause them and their fellow community members?

Members of the patient community—and the foundations which they may participate in, and which fundraise and advocate for more research and better therapies and improved access and greater awareness of patient challenges—benefit more directly than anyone else from societal recognition of the salience of their shared needs through social policy. They are thus personally and financially invested in enhancing the strength, validity, and scope of their claim on society’s resources (whether through government or public-private partnerships or private foundations). Yet this claim rests on the collective nature of their needs, of which the patient community is the embodiment. In this sense, we can conceive of the foundations and affiliated patient organizations and support groups, etc. as the organizational instruments by which patient communities both respond to and influence society, so as to negotiate the collective societal status of community members’ medical needs. This dialectic interaction imposes on the patient community and on the foundations a corresponding ethical responsibility to ensure that care is delivered prosocially—i.e. in such a way as to incorporate the values of transparency, collaboration, equity, and patient empowerment which are a feature of the proposed grant system. Indeed, if the concept of shared, aggregate needs, and a correspondingly shared, aggregate claim on societal resources, is not merely to remain an abstraction, members of the patient community cannot be indifferent to each other’s well-being, or to the resources and accessibility of a system which aims to collectively maximize quality of life in the face of the diseases from which they all suffer. The promotion of a prosocial model thus reifies the very role of the community as a crucial mediator between patient and society. As a result, the community is optimally positioned to repay the claim which it makes on society’s resources, so as to ensure that society gets the best value for its health care spending—in terms of the benefits which flow from government-funded and non-profit research as well as insurance access to commercially developed therapies, which in the case of iPS would be highly expensive and specialized biologics. The measurement of this societal value derives from the effectiveness of society’s *biosocial regulation* of chronic disease. The whole point of this regulation (materialized at the cutting through prosocial distribution of therapies such as disease-specific iPS) is to neutralize the overall social harm of the disease to the greatest extent possible, so as to maximize the potential of the patient community to contribute to the quality and emotional meaningfulness of its members' lives, as well as to the ethical, cultural, and economic welfare of society at large. At the micro-level, such regulation involves minimizing the risk that the disease will interfere with any given patient’s ability to access the career, social, academic, and domestic opportunities available to “healthy” individuals, or to lead an ethically and emotionally fulfilling life. Even apart from the ethical responsibilities flowing from shared needs, the patient community’s familiarity with the medical challenges of its members, without whom it wouldn’t exist, positions it—via its associated organizations and philanthropies—as the most suitable means of facilitating society’s interest in minimizing the health insecurity which stems from chronic disease.

Additionally, patient communities differ both from a demographic which buys a particular consumer product and from communities whose shared interests do not amount to needs. Consider the interests of sports fans or the demographic makeup of plasma TV buyers. A particular plasma TV model, for example, may appeal most to a demographic with empirically measurable characteristics (a particular age range, education level, income distribution, sex, etc.). But these common characteristics relate to ownership of that TV in a way that is merely incidental, since they are only relevant instrumentally for their marketing value (with the ultimate goal being for the manufacturer to sell more units and thus, hopefully, make a larger profit). Ownership of that particular TV model does not constitute a response to a shared threat to quality of life, as would be the case for treatment-seeking among those with the chronic conditions discussed above. Nor, between the owners, does it establish, embody, or modify any political, social or economic connections, expressive of goals or perspectives which would have a salient effect on owners’ social, domestic, or personal identities. But what about a more communal activity such as sports-watching or virtual sports competitions? Even though individuals’ interests in this case might be shared and relevant to social identity, they still likely amount to desires rather than needs. At the very least no commentator has articulated a rational basis for *needing* government subsidies for premium sports channel subscriptions or game tickets. In Wiggins’s formulation, if one *desires* something for a particular reason, that *desire* is based on a *belief* that it will yield a particular benefit; but if one *needs* something, one *needs* it regardless of whether one believes or even knows about its effects [15]. A patient with SCID *needs* a bone marrow transplant, because of the harm that will arise from not having a functional immune system—i.e. an inability to fight off opportunistic infections, followed by likely death before the age of 1 [19].

### Promoting relational autonomy and dignity

Moreover, the more involved, committed, and informed the patient communities, the better patient advocates in decisionmaking roles (e.g. grant-making) will be able to represent patients’ perspectives and ensure that research programs address the concerns that are most clinically relevant to them. This is a critical concern for a prosocial delivery system that aims to maximize its accessibility and empowerment of patients. For example, 12 members of the 29-person governing board of the California Institute of Regenerative Medicine (CIRM) must be patient advocates, and some of these advocates also serve on the CIRM working group that distributes grants for stem-cell research (Sheehy, 2010, p. 1070) [20]. Based on his own experience as a person with HIV, Jeff Sheehy, the vice-chair of CIRM’s grants working group, notes the critical role of his fellow advocates in raising awareness of clinical challenges that are of great concern to patients, but might be overlooked by other board members and scientists. These include many existing therapies’ inadequacy in addressing serious medical and quality-of-life complications in those with chronic conditions (e.g. “increased rates of heart-disease, non-HIV-related cancers and neurological deficits”, in the case of HIV). As Sheehy explains, “When [another CIRM] reviewer opines that he does not deem a proposal worth funding because he doesn’t think anyone with HIV will consider participating in clinical trials of an experimental treatment, I answer that I would, and my voice has changed the discussion” [20]. These patient advocates have shared needs for more effective treatments and more accessible delivery systems in common with other members of their patient communities, and so their very relevance stems from their status as members of such communities—the perspectives they represent and the concerns they raise must be inherently communal, coming from shared aspects of the patient experience which shed a more personal and penetrating light on the most salient clinical obstacles in the way of the best attainable quality of life. Thus the cultivation of more institutionalized and interconnected patient communities, via mechanisms such as the competitive grants proposed here, will facilitate the jobs of patient advocates by expanding patients’ access to information and the very therapies for which the advocates are advocating. The result is likely to enhance the influence and standing of patient advocates, improving their communication with a better-informed and more organized patient community, and thus giving them a more coherent, representative, and popularly legitimate narrative with which to develop and justify strategies for addressing patient needs via grant-making and policy.

To the extent that this proposal might more rationally and effectively target treatments towards patients, improving health outcomes, it would promote beneficence. But it could also promote autonomy, based on bioethicist Shlomo Cohen’s (2013) discussion of the ethical status of “nudging” in health care [21]. According to Thaler & Sunstein, who popularized the concept, a “nudge”, as applied to a given set of choices, is “any aspect of the choice architecture that alters people’s behavior in a predictable way without forbidding any options or significantly changing economic incentives” [21]. Nudging has typically been suggested as a means for promoting economically rational decisionmaking by individuals. Thus my focus on its role in improving relational autonomy—by structuring and expanding opportunities for patients to actively define and re-define the nature of their involvement in collaborative activities such as policy advocacy, provision of feedback to institutions and providers, and participation in clinical trials—may be novel, but it is nonetheless consistent with the limited definition offered above. As direct economic beneficiaries, the institutions that the proposed model would target would not be the nudgees. Rather, the model would incentivize prosocial care delivery systems to nudge good candidates towards greater research participation and iPS treatment. Thus academic medical centers should become more committed to ensuring that their providers access, and then share with patients, information about the broadest relevant range of innovative or experimental treatments and procedures (particularly in life-threatening cases) and clinical trials, including those at other institutions—since centers could still be awarded credit towards grant scores for *referrals*. Additionally, the proposed model aims to strengthen the ties between medical centers and medical foundations (including disease-specific ones that might partner to fund the grants) so as to empower patient communities on multiple levels. We might rationally assume that the majority of patients, particularly those with the severe chronic conditions which my model focuses on, are looking to improve their own health and self-sufficiency, minimize the caretaking burden on their families, and contribute towards research which would extend these benefits across the patient community. Thus an expanded range of treatment options (where medically indicated and practically feasible), and a greater opportunity to become involved in research or advocacy, would likely expand autonomy by “nudging people towards self-proclaimed goals” [21]. And even if certain patients or families have personal or religious objections to participation, this model is in no way coercive, and indeed could reward research ethics and professional oversight boards (IRBs) for developing additional protocols to safeguard patient autonomy. Moreover, a maximally transparent clinical and research agenda, one that focuses on ensuring that patients have optimal information about their therapeutic options, is an *essential precondition for systems* whose goal is to foster autonomous patients who will make decisions according to their own best judgment and values. ^d^ Indeed, referencing the behavioral economics literature, Cohen notes that people tend not even to form definite preferences outside of a given set of choices, so that context and the mode of presentation can determine preferences [21]. Thus medical systems which would aim not only to uphold autonomy *ad hoc*, decision-by-decision, but also to maximize patients’ faith and trust in the system as a whole, would aim likewise to maximize the extent to which patients can become *actively* involved in learning about and managing their own care, so that each patient has a (well-justified) impression that her medical team considers her own concerns and goals of critical importance in formulating a treatment plan.

In a broad sense, this goal is served by reinforcing patients’ sense of empowerment, coming not only from engagement with their own treatment team, but also with the patient community as a whole. Patients should feel they have all the tools possible not only to make a difference in their own care, but also to contribute towards research and policies which will benefit fellow patients undergoing similar struggles. A patient community of people well-informed and confident about their mission is one that is much more valuable to each member, to the advocates who represent them in policy and in grantmaking bodies like CIRM, and to society as a whole—and one in a much better position to achieve the ultimate goal of minimizing the social harm of a particular condition. This argument applies, on the smaller scale of doctor-patient interaction and patient advocacy, one of the primary justifications for the indispensable value of community to each of its members. As philosopher Charles Taylor points out: “the free individual and autonomous moral agent can only maintain his identity in a certain type of culture… carried on in institutions and associations which require stability and continuity and frequently also support from society as a whole” [22]. Perhaps my model is most comprehensively captured by barrister-bioethicist Charles Foster’s (2011) expanded conceptualization of human dignity as a broad-based bioethical value, one that measures the extent to which all interactions in health care promote human flourishing. For Foster, dignity is a state of life which best embodies and perpetuates those qualities which are vitally human, while recognizing our inherent interdependence on each other—encompassing what makes us, as relational beings, thrive at an emotional, moral, physical, and spiritual level, based on empirical insights from medicine and the social sciences [9]. This is a radically holistic concept, and it obviously requires qualification in a particular context. But if anything is dignity-promoting, surely it is a model based on prosocial care delivery and strong patient communities—collectively focused on the flourishing of everyone in society with a given disease, and not only acknowledging but embodying patients’ inherent interconnectedness via their shared needs.

But if the patient community were an unqualified, ethically exclusive aspiration, it might justify unacceptable restrictions on patient autonomy, even to the extent of compelling patients to participate in medical research. As a value, prosociality in healthcare—manifested through collaboration, transparency, patient-empowerment, and equity—must still be balanced by a respect for autonomy. An all-powerful community, one which could serve as a justification for paternalistic coercion, might theoretically intervene in patients’ treatment decisions without limit, creating a medical culture which threatened rather than reinforced patients’ and family caregivers’ sense of safety, personal freedom, and faith in the legitimacy of the care-delivering institution. Thus the proposed model reflects the sort of “libertarian” or “asymmetric” paternalism exemplified through non-coercive nudging [22]. A liberal democratic respect for individual rights would make us deservedly wary of any form of community that could compel patients (or competent caregivers with proxy in the case of children or non-competent adults), even under limited circumstances, to participate in a particular study, or to receive a particular treatment or experimental therapy, even after being fully informed of the nature of the intervention and rejecting it (as philosopher John Ladd points out, an extreme form of coercive communitarianism can even justify fascism) [23]. (Whether there are any conceivable conditions that would justify such compulsion is a question beyond the scope of this paper).

### Patient communities within the public health context

Within the health care context, references to community recur most frequently in the public health literature, but the way the concept is used there differs from the strict definition of a patient community offered here. By distinguishing the two conceptions, however, we may reveal ways that the goals of patients needing highly specialized care may be aligned with the quality-of-life concerns of a more general population. Those working in public health tend to think of community in a looser sense, as what might be called a “public health network”—a more broad-based web of institutions and providers focused on meeting the public health needs of a particular, geo-culturally delineated population, often within a primary-care setting. Such a network might contain diverse ethnic groups, along with some chronically and mentally ill patients, who altogether would have partially overlapping interests, goals, and needs, but the majority of its members’ lives would not be as unavoidably intertwined with the healthcare system as would *necessarily* be the case for members of a patient community. Compared with a patient community, then, such a grouping could not have as uniform or specific a claim of need. However, members of a broader public health network might have very similar policy goals centered around improving quality of life and community participation, often expressed in terms of local issues—improved health education in schools, more funding for community-health centers, more providers who conduct preventive screenings for diabetes or cancer. Thus the very broad-based nature of such a network could draw together a multifaceted range of intersecting interests, serving as a sort of “umbrella” community. And this could create an interface ideally suited for individual patient communities to interact with each other, focus their volunteering efforts, advocate for shared policy goals, and exert more influence on providers and the care delivery system. Indeed, a community public health network, by design and by history, is supposed to be more rhizomatic and receptive to patient feedback [11].

The problem is that such networks have traditionally had far fewer resources than multi-specialty academic medical centers, so that when highly specialized therapies (such as experimental stem cell transplants) are needed to treat congenital or severe chronic diseases, patients have had to resort to such centers as the most viable option for treatment, perhaps subsequently having little incentive to connect with community public health networks focused on primary care. The proposed incentive scheme aims, in part, to encourage academic medical centers to adopt the patient-as-stakeholder mentality to which community health centers (at least ostensibly) aspire, within the context of a range of therapies which cannot be delivered solely at the primary care level.^b^ These groups could offer basic follow-up and monitoring, health education—regarding symptoms and best practices for avoiding complications and comorbidities, and for communicating effectively with providers—as well as referral to the academic centers (with which they might or might not be institutionally affiliated) for more specialized care, an arrangement which might prove particularly useful for lower-income patients. Moreover, the stronger, more engaged patient communities which my proposal seeks to foster would be better positioned to partner with other stakeholders within the community public health network, so as to maximize their overall influence on health-care decisionmaking and local quality-of-life issues. Indeed, the proposal is broadly sympathetic to proposals for community-based participatory research, but would seek to extend its scope to specialized care delivery, expand its aims in terms of patient involvement in policy advocacy and institutional decisionmaking, and ideally try to promote partnership between community health practices and academic medical centers. NIH, for example, has already created a network of “breast cancer and the environment research centers”, involving collaboration between “scientists, advocates, community members, and health care providers” [24].

A similar arrangement, eliciting more direct participation from academic medical centers, and covering more specialized chronic conditions such as those which are the subject of iPS research, might allow for a more complete systematization of patient participation and influence, one which harnessed biomedical in addition to social science innovation. Within such a context, incentivizing partnerships between community health networks and academic centers might also help to alleviate the networks’ traditional difficulty in raising private funding, given the tendency of domestic foundations and wealthy donors to focus on groundbreaking scientific innovation as opposed to primary care and prevention. Indeed, if patient communities could achieve greater policy impact and more influence over medical decisionmaking by participating in community health networks, then disease-oriented foundations interested in the welfare of patient communities might also have more incentive to focus on strengthening such networks.

### Ethical foundations and the limits of “capability”: response to criticism

The model proposed here, aimed at minimizing the harm of chronic conditions by promoting a holistic, relational concept of quality of life—encompassing a broad range of criteria, including health status, access to information, opportunities for education, work, and recreation, as well as the quality, variety and personal meaningfulness of social interaction—which altogether would facilitate human flourishing, may resemble and complement the “capabilities approach” advocated by Nussbaum and Sen, applied specifically to the context of patient experience by Entwistle and Watt [25]. This argument does not deny such an overlap or the potential synergies, in policy and in improved, relational metrics for quality of life, which may result from it. However, it is important to emphasize the instrumental rather than the *a priori* nature of these synergies. Contrary perhaps to Nussbaum, my proposal does not suggest that even an expanded conceptualization of personal agency (e.g. one involving emotional and interpersonal well-being as well as rational decisionmaking capability) should be the ultimate ethical aspiration. A more promising *a priori* basis for dignity, one based on the inherent ethical potential of life itself, may lie in a Levinasian concept, whereby the Self’s own corporeality places it in infinite ethical subjection to the Other (a formal category which, as Judith Butler puts it, serves as “a placeholder for the infinite ethical relation”) [26,27]. A discussion of meta-ethics, of what it means to respect the Other’s radical alterity—of why one should have an obligation to act ethically without ignoring the Other’s infinite demandingness, whose trace a static ontologized self-concept or worldview would disparage—is beyond this paper’s scope. But such an aspiration (whose infinitude would render it un-dischargeable and ultimately unfulfillable) would suggest that, in making ethical arguments, it is the inherent quality of *ethical relations themselves* that count. This relational quality, the assessment of which is inevitably context-dependent, may in most instances be enhanced by following the capability approach, but the danger of following the capability approach *exclusively* is that it might undermine the ethical value of relationships which involve caring for someone with little or no capacity for agency, e.g. patients who have severe dementia or anencephaly, or are in a persistent vegetative state (PVS).

Indeed, many cultures do not share the Western notion of individual autonomy, and this article’s focus on the way that relational interaction can enhance and even create the conditions for autonomy is partly an attempt to expand the global applicability of the concept. The mitigation of coercive, top-down interference by some people and institutions in the lives of others, at both an individual and a cultural level, would allow communities of individuals to form and evolve temporally—without being locked into a particular pattern of interaction which cannot keep pace with cultural development, and which accordingly may come to restrict opportunities for beneficent action. Such a goal, expressed through a concept such as relational autonomy (which in this argument is not supposed to imply a permanent, unvarying method of achieving the goal) is expansive enough to encompass a wide variety of different cultures. The prosocial principles from which the grant criteria derive are admittedly instrumental and culturally contingent, but they are still the best means of upholding a relational conception of human dignity vis-à-vis the clinical translation of medically innovative therapies for patients with chronic diseases. Like autonomy itself, they are not ends in themselves, but that does not mean that they still aren’t ethically preferable or even obligatory within most contexts.

Moreover, the aim of making advocacy more rhizomatic and participant-directed (as described below), e.g. through the vehicle of patient communities, does not necessarily mean that the resources for such advocacy must be exclusively provided by the participants, those who belong to the particular community with the relevant ethical claim. A patient community may exist, in the ethical sense discussed here, whether or not that community has resources of time and information and money to pursue its goals, but that does not mean that the provision of resources top-down, e.g. by a foundation or medical center or government body not primarily managed and funded by patients, will necessarily distort the achievement of such goals. (Of course, many of the people who fund and manage disease-specific foundations do indeed have a family member affected by the disease, and some may even suffer from it themselves). Some concern about conflicts of interest is of course legitimate, but this can be minimized through funding mechanisms designed to preserve impartiality. Indeed, social inertia and a structural lack of resources—of information and money and manpower—will likely serve only to marginalize the societal influence of the very patients who constitute patient communities, outweighing the risk that top-down funding may hinder patients from expressing their views in a manner of their choosing, or may force them to conform to one particular policy perspective. The relevant concern is in *the nature of the procedures by which the community operates*—do they promote a lateral, non-hiearchical structure that is optimally positioned to pursue its aims of patient empowerment within an evolving cultural context?

In the end, there is one overriding ethical and methodological theme to my discussion of a prosocial delivery system and a patient community based on shared needs. While evaluating the circumstances of individual cases, bioethicists may too often focus on patients and families in isolation from the prevailing cultural and political context in which the relevant problems occur, and may pay too little heed to the mutually reinforcing potential of patient interaction and advocacy. Although government regulatory and institutional policies constitute the framework for individual cases, such policies should themselves be more responsive to patient input. Indeed, the medical and regulatory context should be considered through the lens of the collective societal status of patient groups—not merely as a set of procedures shaped by policymakers in a one-way, top-down process, but as offering the potential for a dynamic interaction with patient communities. Of course, major legislation (e.g. the Affordable Care Act) does impose certain top-down limits, defining socio-political paradigms which cannot be fundamentally altered without new laws (e.g. employment-based insurance in the U.S.). But the resources and social influence of medical philanthropies and academic medical centers within the non-profit sector, as well as regulatory bodies' discretion and reliance on expert input, along with their potential for greater patient inclusion (e.g. in the case of CIRM), may, regardless of new legislation, allow for the adoption of shifts in patient-provider and patient-society interaction which are more rhizomatic—affording opportunities for lateral, less hierarchical, and more closely intertwined channels of patient feedback which can exert meaningful influence on patient care. Thus stronger patient communities may not only enhance patient autonomy by facilitating a more active, informed patient role in treatment decisionmaking. They may also give patients the social clout to become a more important and less easily ignored stakeholder group in the governing of the care delivery system which affects them more personally than anyone else.

## Summary

"Community" is one of the most salient buzzwords in the public health and health policy literature, but the ethical basis and implications of the concept of a patient community have remained largely unexplored. This article’s grant proposal aims to spur innovation in *delivery* of care for patients with chronic conditions which are subjects of disease-specific stem cell research, so as to contribute to a more systematic, accessible and patient-influenced delivery model, reifying the relevance of community via values of transparency, equity, patient empowerment, and patient-provider and inter-institutional collaboration. There is a special focus on disease-specific iPS cell therapies because the types of patients who would benefit from them have congenital, severe, high-maintenance chronic conditions. They accordingly have a very strong claim for medical need and therapeutic intervention, must necessarily interact regularly with health providers, and so have the greatest stake in influencing, at a *systemic level*, the way their care is delivered.

Moreover, the clinical testing of these therapies offers an opportunity to design a new model for delivery of medically innovative treatments to patients with chronic conditions. Such a *prosocial* system is ethically premised on the recognition of patients’ shared, aggregate *needs* for societal support and access to medical innovation. While this requirement of aggregate, shared, and consistent need strictly defines the patient community, I in no way intend to introduce a concept in contention with the kind of broad-based, geo-culturally embedded public health network which the public health literature typically refers to as a “community”. Indeed, public health networks can serve as an interface through which strictly defined patient communities can interact with more broad-based groups or coalitions, advocating together for health and quality-of-life-improving measures.

The discussion concludes by explaining the aims of prosocial values—to not only expand treatment choices and opportunities for patients to take a more active role in the management of their own care, but also to contribute towards shared patient goals through better-informed advocacy. Such values accordingly promote relational autonomy as well as other basic bioethical principles, including beneficence and a holistic, relational conception of human dignity.

## Endnotes

^a^Therapeutically, the goal is to be able to safely transplant reprogrammed cells back into the patient from whom they were sourced, avoiding the risk of immune rejection associated with transplants between unrelated donors [5]. Disease specific stem cells can either be sourced from embryos identified as carrying a disease through pre-implantation genetic diagnosis (embryonic stem cells—ES), or they can be developed by taking fibroblasts (a basic connective tissue cell) from individuals with the disease and transducing them with transcription factors which confer stem cell characteristics (producing iPS—induced pluripotent stem cells) [17]. Researchers now prefer iPS cells because they can more effectively model genetically complex diseases, and because disease-specific ES cells have limited availability and can be logistically inconvenient to obtain [17].

^b^But far from cutting community health centers out of the picture, grants might incentivize academic medical centers to partner with primary-care focused community health groups. The “collaboration” criterion of my proposal, however, would not be so broad-based as to cover such partnerships, so an additional grant scheme would be needed.

^c^Medicaid is a joint federal-state program for indigent and disabled patients below certain income thresholds (set at 133% of the federal poverty limit in states which have accepted the additional Medicaid funding made available through the Affordable Care Act).

^d^Nudging is typically recommended as a means for correcting irrational biases, in which individuals weigh the utility of a given choice differently than its objective, quantitatively determined value, resulting in a decisionmaking “error”. The classic example is loss aversion, “where the negative value people assign to a given loss is larger in absolute terms than the positive value they assign to the identical gain” (Cohen 2013, 8). My proposal could of course incorporate such insights, but only to the extent they help expand the quantity, quality, and relevance of the information available to consenting patients who wish to take a more active role in the management of their care.

## Abbreviations

CIRM: California Institute of Regenerative Medicine; iPS Cells: induced pluripotent stem cells; NIH: U.S. National Institutes of Health; IRB: Institutional (Research Ethics) Review Board; ISSCR: International Society for Stem Cell Research; PVS: Persistent vegetative state; SMA: Spinal muscular atrophy; SCID: Severe combined immunodeficiency syndrome.

## Competing interests

The author declares that he has no competing interests.

## Pre-publication history

The pre-publication history for this paper can be accessed here:

http://www.biomedcentral.com/1472-6939/15/16/prepub
